# Supplementary material for: Binary dopant segregation enables hematite-based heterostructures for highly efficient solar H2O2 synthesis
Source: Nat Commun. 2022 Mar 23;13:1499. doi: 10.1038/s41467-022-28944-y (PMC8943161; doi:10.1038/s41467-022-28944-y)
Supplement: Supplementary file 1 — Supplementary Information [file 41467_2022_28944_MOESM1_ESM.pdf]

Supplementary Information

Binary dopant segregation enables hematite-based heterostructures for highly efficient solar H_2O_2 synthesis

Zhujun Zhang[1], Takashi Tsuchimochi[2,3], Toshiaki Ina[4], Yoshitaka Kumabe[1], Shunsuke Muto[5], Koji Ohara[4], Hiroki Yamada[4], Seiichiro L. Ten-no[2,6], and Takashi Tachikawa[1,7]*

[1]Molecular Photoscience Research Center, Kobe University, 1-1 Rokkodai-cho, Nada-ku, Kobe 657-8501, Japan

[2]Graduate School of System Informatics, Kobe University, 1-1 Rokkodai-cho, Nada-ku, Kobe 657-8501, Japan

[3]PRESTO, Japan Science and Technology Agency (JST), 4-1-8 Honcho Kawaguchi, Saitama 332-0012, Japan

[4]Japan Synchrotron Radiation Research Institute, 1-1-1 Kouto, Sayo-cho, Sayo-gun, Hyogo 679-5198, Japan

[5]Electron Nanoscopy Section, Advanced Measurement Technology Center, Institute of Materials and Systems for Sustainability, Nagoya University, Furo-cho, Chikusa-ku, Nagoya 464-8603, Japan

[6]Graduate School of Science, Technology, and Innovation, Kobe University, 1-1 Rokkodai-cho, Nada-ku, Kobe 657-8501, Japan

[7]Department of Chemistry, Graduate School of Science, Kobe University, 1-1 Rokkodai-cho, Nada-ku, Kobe 657-8501, Japan

*Correspondence and requests for materials should be addressed to T.T. (e-mail: tachikawa@port.kobe-u.ac.jp).

This PDF file includes:

Supplementary Notes 1–4

Supplementary Figs. 1–32

Supplementary Table 1–5

Supplementary Note 1

The mechanism of the dopant segregation induced by electrostatic interactions to compensate ionic space charges at (sub)surface and/or grain boundaries (GBs) has been well established.[1–4] In this note, we summarize the main part of the space-charge theory related to our study.

Here, Fe_2O_3 is assumed to be composed of trivalent metal cations, Fe^{3+}. It is also assumed that the Fe_2O_3 crystal has a free surface and GB (x = 0). The predominant lattice defects are considered as Schottky-type defects and showed based on the Kröger–Vink notation. The concentrations of vacancies for iron cation ($[\mathrm{V}_{\mathrm{Fe}}^{\prime\prime\prime}](x)$) and oxygen anion ($[\mathrm{V}_{\mathrm{O}}^{\bullet\bullet}](x)$) and interstitial iron ($[\mathrm{Fe}_{\mathrm{i}}^{\bullet\bullet\bullet}](x)$) are described as

$$[\mathrm{V}_{\mathrm{Fe}}^{\prime\prime\prime}](x) = \exp\left(-\frac{(g_{\mathrm{V_{Fe}}} - 3e\phi(x))}{kT}\right) \tag{1}$$

$$[\mathrm{V}_{\mathrm{O}}^{\bullet\bullet}](x) = \frac{3}{2}\exp\left(-\frac{(g_{\mathrm{V_{O}}} + 2e\phi(x))}{kT}\right) \tag{2}$$

$$[\mathrm{Fe}_{\mathrm{i}}^{\bullet\bullet\bullet}](x) = \exp\left(-\frac{(g_{\mathrm{Fe_{i}}} + 3e\phi(x))}{kT}\right) \tag{3}$$

The formation free energies $g_{\mathrm{V_{Fe}}}$, $g_{\mathrm{V_{O}}}$, and $g_{\mathrm{Fe_{i}}}$ are defined relative to an interface. The ϕ_∞ denotes the electrostatic potential in the bulk far from the surface or GB and can be calculated by using the charge neutral condition, i.e., $3[\mathrm{V}_{\mathrm{Fe}}^{\prime\prime\prime}]_\infty = 2[\mathrm{V}_{\mathrm{O}}^{\bullet\bullet}]_\infty = 3[\mathrm{Fe}_{\mathrm{i}}^{\prime\prime\prime}]_\infty$. Then, we can obtain

$$e\phi_\infty = \frac{1}{6}\{g_{\mathrm{V_{Fe}}} - g_{\mathrm{Fe_{i}}}\} \tag{4}$$

$$e\phi_\infty = \frac{1}{5}\{g_{\mathrm{V_{Fe}}} - g_{\mathrm{V_{O}}}\} \tag{5}$$

According to equations (4) and (5), the electrostatic potential in the bulk has a relatively low value for pure hematite. However, the potential in the space-charge layer will be remarkably modified by doping aliovalent cations to the lattice and thus provide a potential gradient to drive the dopant segregation in the space-charge layer.

For example, in the case of the Ti^{4+}-doped Fe_2O_3 system, Ti^{4+} is added as a donor in Fe_2O_3. To reach the charge neutrality in the bulk, $\mathrm{V}_{\mathrm{Fe}}^{\prime\prime\prime}$ will form. The concentration of $[\mathrm{V}_{\mathrm{Fe}}^{\prime\prime\prime}]$ is readily determined by the condition $[\mathrm{Ti}_{\mathrm{Fe}}^{\bullet}]_\infty = 3[\mathrm{V}_{\mathrm{Fe}}^{\prime\prime\prime}]_\infty$. Therefore, the electrostatic potential between the bulk and surface can be deduced as equation (6),

$$e\phi_\infty = \frac{g_{\mathrm{V_{Fe}}}}{3} + \frac{kT}{3}\ln\frac{[\mathrm{Ti}_{\mathrm{Fe}}^{\bullet}]_\infty}{3} \tag{6}$$

Consequently, in the donor-doped case, the ϕ_∞ has a large value with positive sign, and thus the electrostatic potential in the space-charge layer ($\phi(x)$) rapidly decays. According to equation (1), the concentration of iron vacancies ($[\mathrm{V}_{\mathrm{Fe}}'''](x)$) in the space-charge layer is significantly reduced and thus leads to the strong accumulation of positively charged $\mathrm{Ti}_{\mathrm{Fe}}^{\bullet}$, which should be compensated by negative charges (e.g., $\mathrm{O}_{\mathrm{sup}}''$) on the surface (Fig. 1a). It was recently found by the atom probe tomography analyses that doped titanium cations in hematite were segregated to the O-rich grains on the surface,[5] which is consistent with our finding that annealed Ti-Fe_2O_3 possesses more negative potentials on the edge (i.e., surface) as revealed by Kelvin prove force microscopy (KPFM) measurements (Supplementary Fig. 2).

For the acceptor doping case, i.e., Sn^{2+}-doped Fe_2O_3, the electrostatic potential can be also derived in the same manner. The effective negative charges by substituted tin anions will be compensated by oxygen vacancies via the relationship of $[\mathrm{Sn}_{\mathrm{Fe}}']_\infty = 2[\mathrm{V}_{\mathrm{O}}^{\bullet\bullet}]_\infty$. The electrical potential for the acceptor doping case in the bulk is therefore given as

$$e\phi_\infty = -\frac{g_{\mathrm{V_O}}}{2} - \frac{kT}{2}\ln\frac{[\mathrm{Sn}_{\mathrm{Fe}}']_\infty}{2} \tag{7}$$

In contrast to the donor doping case, the ϕ_∞ has a large value with negative sign (Supplementary Fig. 1), which contributes to the acceptor segregation in the space-charge layer.

Based on the above consideration, the concentration of the dopants can be generally expressed as the exponentially varying simple Boltzmann distribution.

$$\frac{n_i(x)}{n_{i,\infty}} = \exp\left\{\frac{-z_i e[\phi(x)-\phi_\infty]}{kT}\right\} \tag{8}$$

where n_i and z_i are the concentration and effective charge of the dopant, respectively. It is clear that dopant segregation is driven by the electrostatic potential in the space-charge layer. Therefore, the increase of the electrical potential in the space-charge layer can provide a larger potential to promote the dopant segregation.

During thermal annealing of hematite MCs, oxygen vacancies are formed at the interfaces along with electrons as described by the following defect reaction

$$\mathrm{O}_{\mathrm{O}}^{\times} \rightleftarrows 2\mathrm{e}' + \mathrm{V}_{\mathrm{O}}^{\bullet\bullet} + \frac{1}{2}\mathrm{O}_2(\mathrm{g}) \tag{9}$$

where $\mathrm{O}_{\mathrm{O}}^{\times}$ is an oxygen ion at an oxygen site. The electrons are instantaneously captured by Fe^{3+} to form Fe^{2+} as

$$\mathrm{Fe}_{\mathrm{Fe}}^{\bullet\bullet\bullet} + \mathrm{e}' \rightleftarrows \mathrm{Fe}_{\mathrm{Fe}}' \tag{10}$$

where Fe'_{Fe} is Fe^{2+}. Considering the electronic conduction of hematite is a result of polaron hopping, [678] the formation of Fe^{2+} sites can improve the electrical conductivity, especially near the GB. Furthermore, the positively charged V_O localized at the GB core provides an additional repulsive force against the dopant cations to accelerate the oriented migration of the dopant cations from the bulk to the surface without their accumulation to the GB regions.

Supplementary Note 2

The width of space charge layer (W) can be calculated according the following equation,

$$W = \sqrt{\frac{2\varepsilon\varepsilon_0 V_{\mathrm{bi}}}{eN_{\mathrm{d}}}} \tag{11}$$

where ε is the dielectric constant of hematite (80), V_{bi} is the built-in potential which can be calculated by subtracting the flat-band potential from the applied potential, N_d is the carrier density. Based on equation (11), the value of W is dependent on N_d. The N_d value can be calculated according to the following equation,

$$N_{\mathrm{d}} = \frac{2}{e\varepsilon\varepsilon_0}\left[\frac{\mathrm{d}(1/C^2)}{\mathrm{d}V}\right]^{-1} \tag{12}$$

where $\mathrm{d}(1/C^2)/\mathrm{d}V$ is the slop of the obtained Mott-Schottky curve.

The carrier density (determined by the Mott-Schottky curves in Supplementary Fig. 13) of annealed MC samples (~10^{20} cm^{-3}, Supplementary Table 1) are almost 10 times higher of the randomly aggregated hematite single crystals (SCs) (~10^{19} cm^{-3}) without interfacial V_O,[9] and the W value can thus effectively decrease from ~20 nm to below 10 nm in the MC system. This can significantly increase the potential gradient to drive the charge migration for efficient water oxidation.[9]

Supplementary Note 3

For 1/12 ML coverage (Terminal site), the computed ΔG_{OH*} values were 0.62 and 1.33 eV, without and with U, respectively (Supplementary Fig. 25 and Supplementary Table 2). This increase is in fact the same trend as has been discussed by Xu et al.;[10] namely, introducing the Hubbard correction generally weakens adsorption. Furthermore, the latter value is in good agreement with the previously reported value (~1.5 eV),[11] which was obtained by using PBE + U (U = 4.3 eV) and 1/12 monolayer (ML).

With ΔG_{O*} = 3.45 eV computed with U, which also agrees well with the above reference,[11] the O_2 evolution reaction is predicted to dominate over the H_2O_2 evolution reaction in Fe_2O_3.

As soon as the coverage increases from 1/12 ML, we found a large increase in ΔG_{OH*} with $U = 4.3$ eV to 1.74–1.88 eV on average. The computed ΔG_{OH*} for 2/12 ML was found to be 1.74 eV on average, indicating a free energy of 2.15 eV is required to further attach the second OH to the 1/12 ML surface. This is an important insight, because it is strongly indicated that this step has a large thermodynamic barrier and is unlikely to occur at an extra bias of 1.76 eV, ideal for the H_2O_2 evolution reaction. These results also support the experimental evidence that Fe_2O_3 is an O_2 evolution catalyst (Supplementary Fig. 26).

Supplementary Note 4

The production of H_2O_2 via water oxidation reaction (WOR) is a two-electron process with a standard redox potential ($E°$) of 1.76 V, which is 0.53 V higher than that for O_2 generation from four-electron reaction (equations (13) and (14)).

$$2H_2O \rightleftarrows O_2 + 4H^+ + 4e^- \qquad E° = 1.23 \text{ V vs. RHE} \qquad (13)$$

$$2H_2O \rightleftarrows H_2O_2 + 2H^+ + 2e^- \qquad E° = 1.76 \text{ V vs. RHE} \qquad (14)$$

The production of H_2O_2 is a formidable challenge because the two-electron pathway is thermodynamically less favorable than O_2 evolution via four-electron WOR. It requires the two-hole transfer from the bulk to the surface of photoanode semiconductor to activate the PEC WOR for H_2O_2 production from the electrolyte.

In 2016, Sayama's group reported that the PEC H_2O_2 performance of a $BiVO_4/WO_3$ photoanode can be significantly enhanced in HCO_3^- electrolyte.[12] Furthermore, they found that different overlayers of SiO_2, ZrO_2, TiO_2, and Al_2O_3 can increase the PEC H_2O_2 production performance of the $BiVO_4/WO_3$ photoanode in the presence of HCO_3^- electrolyte.[13] According to their explanation, the weak acidic sites on the overlayer surface more effectively adsorb the weakly basic HCO_3^-. HCO_3^- (CO_3^{2-}) acts as a hole acceptor and can be oxidized to unstable HCO_4^- ($CO_3^{\bullet -}$ or $C_2O_6^{2-}$),[14] which then quickly reacts with H_2O to produce H_2O_2 as follows

$$HCO_3^- + H_2O \rightleftarrows HCO_4^- + 2H^+ + 2e^- \qquad E° = 1.76 \text{ V vs. RHE} \qquad (15)$$

$$HCO_4^- + H_2O \rightleftarrows H_2O_2 + HCO_3^- \qquad (16)$$

Nørskov and Zheng et al. proposed a theory of the free energy for absorbed species toward H_2O_2 evolution, O_2 evolution, or hydroxyl radical (•OH) formation via electrochemical oxidation of water. In general, the ΔG_{OH*} determines the number of holes transferred from the surface of metal oxides.[15] Therefore, the ΔG_{OH*} values calculated for the photoanodes can be

used to explain the different amounts of WOR products via the different reaction pathways. Based on their calculation, electrocatalysts for selective H_2O_2 evolution have the G_{OH*} values between ~1.6 and 2.4 eV. The trend in theoretical limiting potentials for WO_3, $BiVO_4$, SnO_2, and TiO_2 are in agreement with their onset potential trend obtained from the experiments. $BiVO_4$ achieved the highest FE of 70% for EC H_2O_2 production at the bias of 3.1 V vs. RHE in HCO_3^--containing electrolyte (pH = 8.3). However, the FE for EC H_2O_2 production was significantly reduced to ~10% when measured the same electrode in 0.5 M Na_2SO_4 at the same pH (adjusted with NaOH). This result suggests that HCO_3^- actually plays an important role to improve the selectivity for H_2O_2 evolution. In addition, the optimal FE of EC H_2O_2 evolution was obtained only under the bias of 3−3.1 V vs. RHE, which might be due to reaction equilibrium between H_2O_2 and O_2 evolution, as also mentioned elsewhere.[16] Compared to the EC H_2O_2 evolution, the activity trends of the PEC H_2O_2 evolution are less dependent on the bias potential since the energy level for PEC WOR is determined by the photoinduced holes. For example, Zheng's group reported that the FEs of over 90% for PEC H_2O_2 evolution can be realized by a Gd-doped $BiVO_4$ electrode under a wide bias potential range of 1.8−2.5 V vs RHE,[17] whereas the FE of EC H_2O_2 for the same electrode was only 78% at 3.1 V vs RHE in HCO_3^--containing electrolyte. When measured the same electrode in other electrolytes (in the absence of HCO_3^-), PEC H_2O_2 production was undetectable. To study the influence of electrolytes, we evaluated the PEC H_2O_2 production by the SnTi-Fe_2O_3 photoanodes in different electrolytes such as phosphate buffer solution (pH ≈ 8.0) and NaOH solution (1.0 M, pH = 13.6). As shown in Supplementary Fig. 31, the onset potentials measured in phosphate buffer solution and NaOH solution are much lower than that measured in $NaHCO_3$ solution. In addition, gaseous oxygen was linearly generated in these electrolytes, suggesting the prioritized water oxidation to O_2 via the four-hole process without the presence of HCO_3^- .

Supplementary Figures

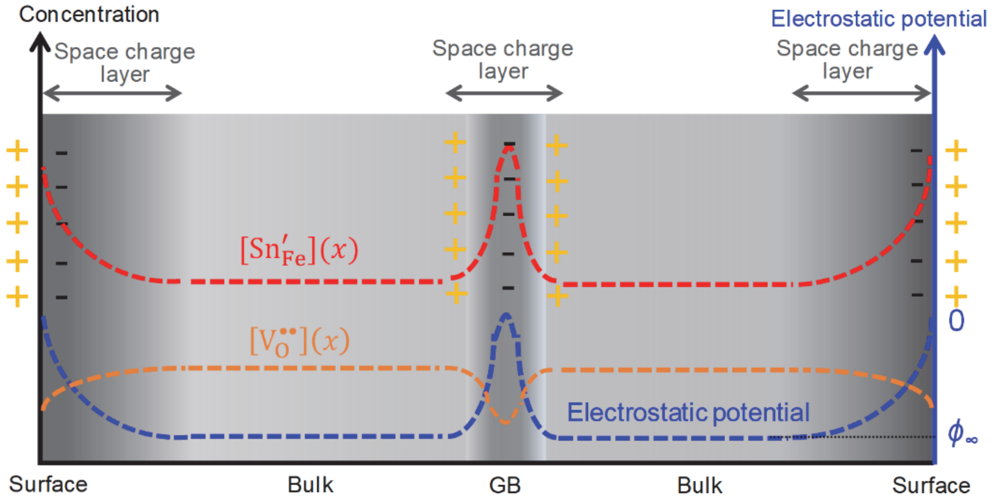

Supplementary Fig. 1. Distribution of dopants and electrostatic potential (acceptor-doping case). Distribution of doped Sn^{2+} cations (Sn'_{Fe}) and oxygen vacancies ($V_O^{\bullet\bullet}$) and electrostatic potential in ionic oxides based on the space-charge theory. See Supplementary Note 1 for details.

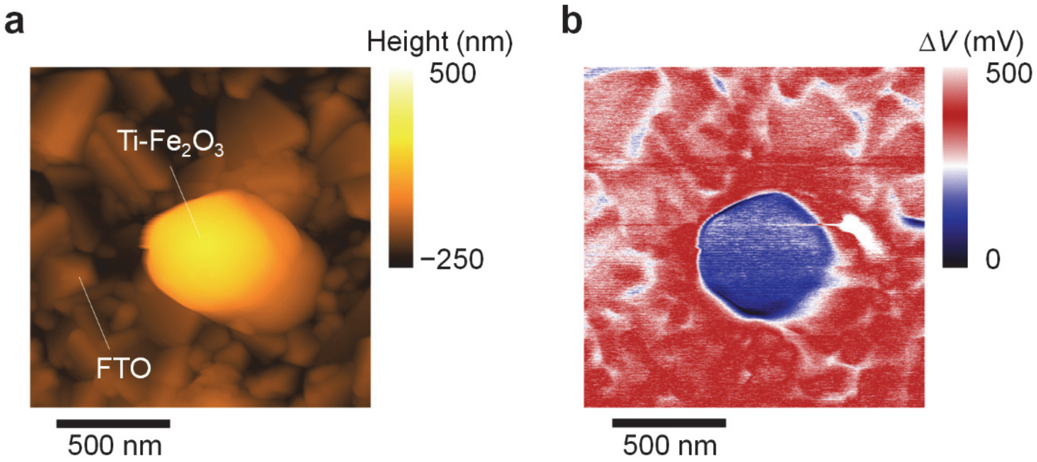

Supplementary Fig. 2. AFM and KPFM measurements. AFM topological (**a**) and KPFM (**b**) images of annealed Ti-Fe_2O_3 on the FTO surface. The potential difference (ΔV) was obtained relative to the probe. The preparation of the sample was reported in our previous paper.[9] From the panel **b**, it can be seen that the edge (i.e., surface) of the particle exhibits more negative potentials.

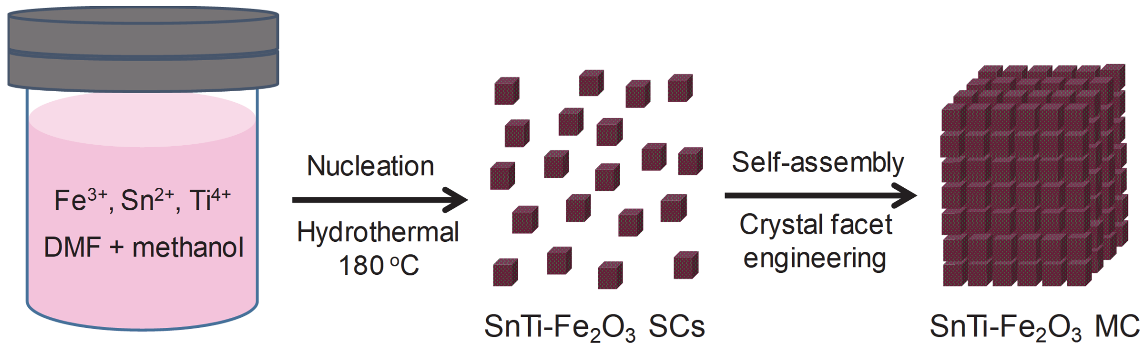

Supplementary Fig. 3. Schematic illustration of the synthesis of SnTi-Fe_2O_3. The hematite MCs containing Sn^{2+} (6.5 mol%) and Ti^{4+} (6.5 mol%) dopants were synthesized via a simple surfactant-free solvothermal method.

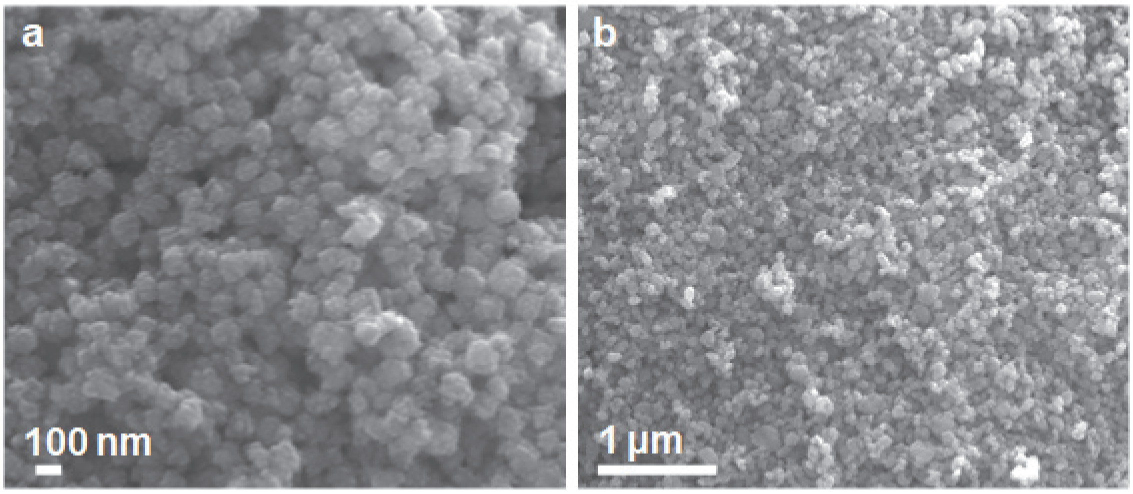

Supplementary Fig. 4. FE-SEM images of the as-synthesized SnTi-Fe_2O_3. a, High-magnification image. **b**, Low-magnification image.

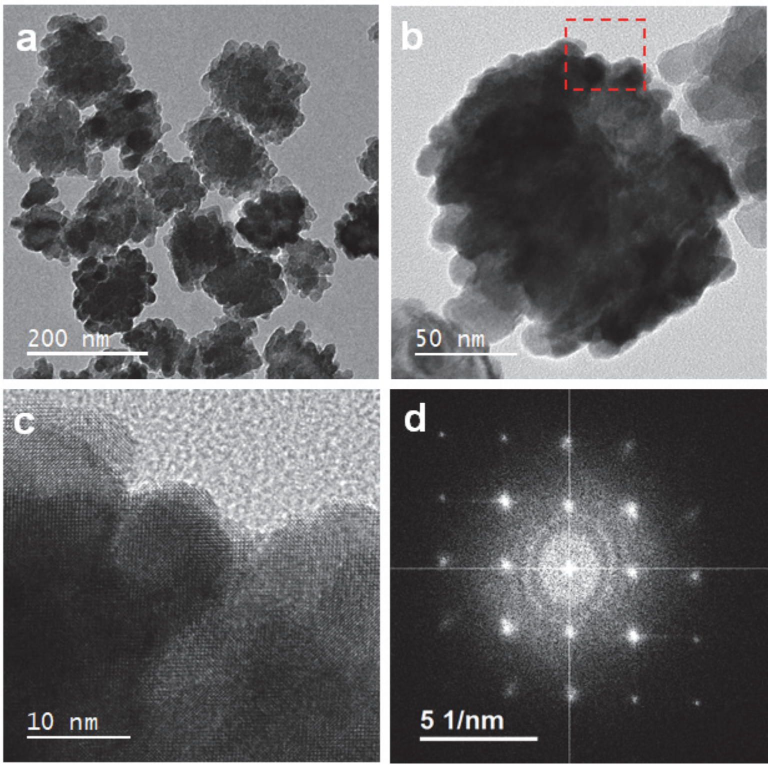

Supplementary Fig. 5. TEM analysis of the as-synthesized SnTi-Fe_2O_3. (**a**, **b**) TEM images of the as-synthesized SnTi-Fe_2O_3. HRTEM image (**c**) and the corresponding FFT pattern (**d**) of the selected area in panel **b**, which shows adjacent nanocrystal subunits inside MC.

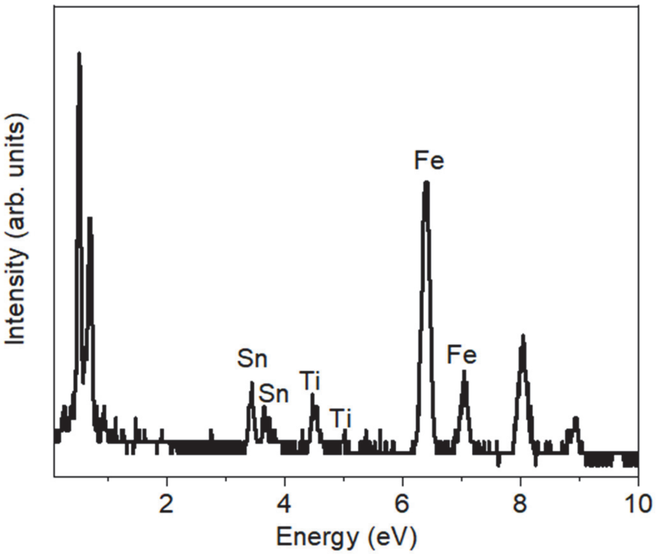

Supplementary Fig. 6. EDX spectrum of the annealed SnTi-Fe_2O_3. The spectrum indicates the peaks corresponding to Ti and Sn in addition to Fe.

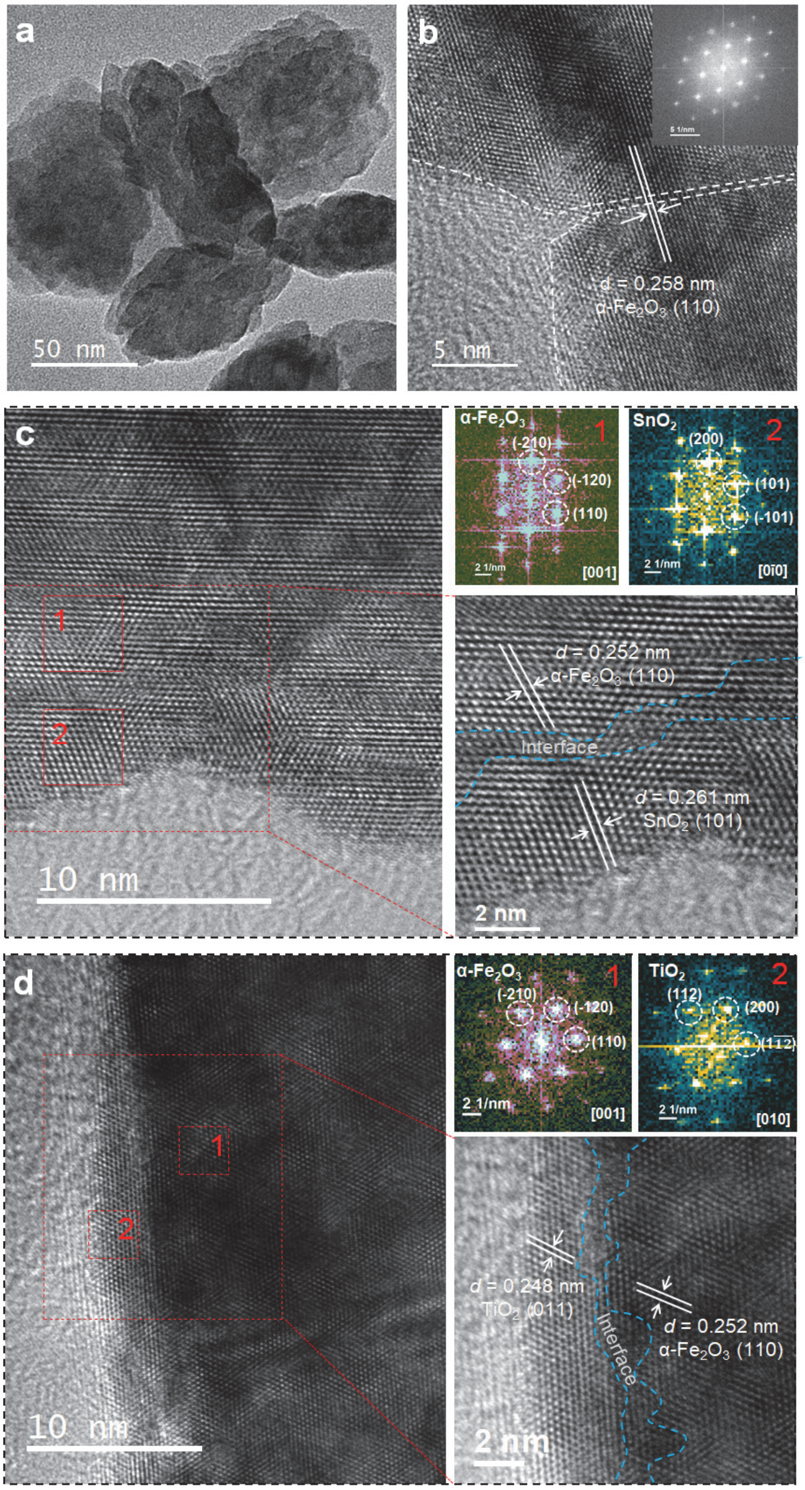

Supplementary Fig. 7. TEM analysis of Sn-Fe_2O_3. TEM image (**a**) and HRTEM and corresponding FFT (**b**) images of as-synthesized Sn-Fe_2O_3. **c**, HRTEM image and corresponding FFT images of the annealed Sn-Fe_2O_3. **d**, HRTEM images and corresponding FFT images of the annealed Ti-Fe_2O_3.

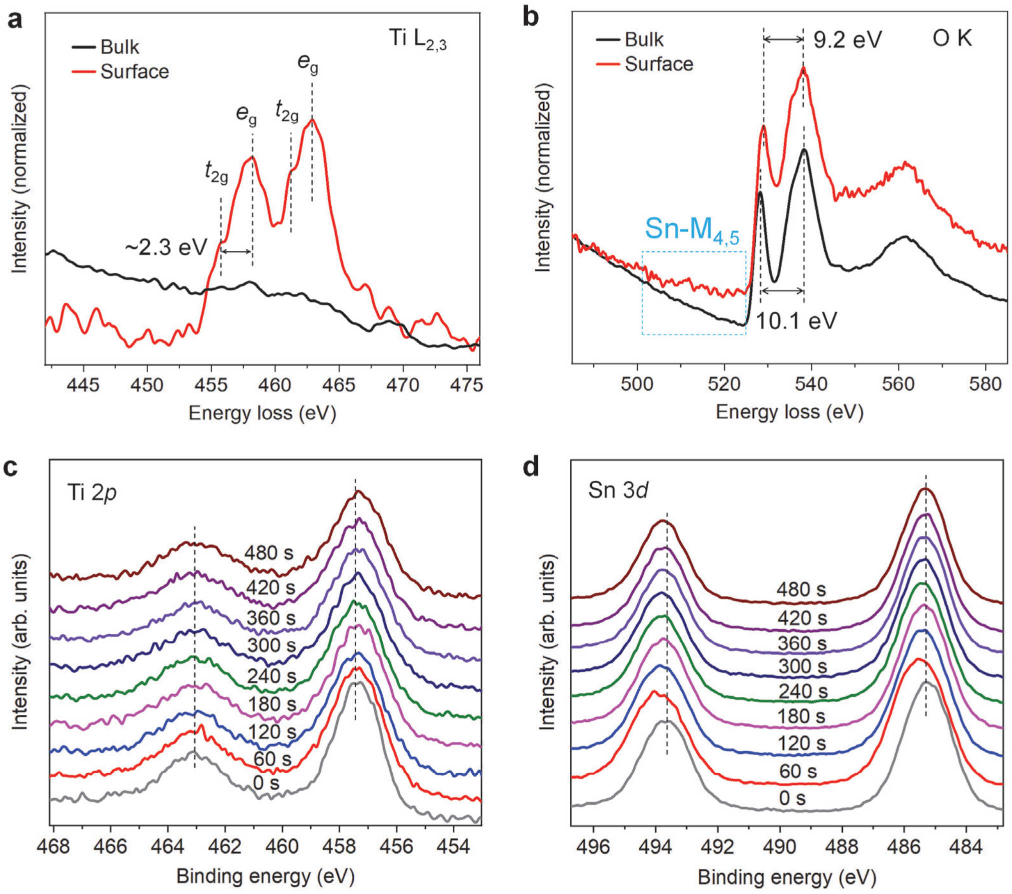

Supplementary Fig. 8. STEM-EELS and XPS measurements. The corresponding EELS Ti-$L_{2,3}$ (**a**) and O-K (**b**) spectra of the selected region in the Fig. 2e. Ti 2*p* (**c**) and Sn 3*d* (**d**) XPS depth profiles of the annealed SnTi-Fe_2O_3 measured by Ar etching for different times. The Ti-$L_{2,3}$ edge is split into two doublets (Supplementary Fig. 8a), which is due to the energetic splitting of t_{2g}- and e_g-orbitals in the TiO_6-octahedral crystal field.[18] The crystal field splitting measured at the $P_{3/2}$ level (~2.3 eV) is close to that reported for ilmenite-type $SnTiO_3$ structure (~2.4 eV).[19] In the O-K edge (Supplementary Fig. 8b), the ionized edges at around 510–520 eV are corresponding to the M_4 and M_5 delayed edges of Sn, respectively.[20] The O-K edge is split into two main signals at 538.2 and 529.0 eV for the surface region, and 538.3 and 528.2 eV for the bulk region, respectively. The first peak, which represents the existence of oxygen, is much lower than the second peak related to the hybridized transition between oxygen and neighbored metal ions, thus suggesting the presence of V_Os in both the surface and bulk regions.[21] The energy difference between these two peaks for the bulk region (~10.1 eV) is very close to the reported value of pure hematite,[22] while that for the surface region (~9.2 eV) is much lower (i.e., the formation of heterostructure). This value is also larger than those reported for tin oxides (4.2–6.3 eV) and titanium oxides (2.5 eV),[18] thus excluding the

possibility of the formation of single-metal oxides as the final main products on the surface. However, we noticed that the Sn 3*d* XPS depth profiles show that the Sn 3*d* signals slightly shifted to higher binding energies when the sample was etched by Ar for 60–120 s (i.e. 0.83–1.66 nm depth) (Supplementary Fig. 8d), while Ti ions possess the same oxidation state from the surface to the depth of ~7 nm as indicated by the non-shifted Ti 3*d* signals (Supplementary Fig. 8c). These results suggest the possibility of existing oxidized Sn ions at the outer surface region (below 2 nm depth). The Sn 3*d* peaks located at relatively lower energies observed before Ar etching are probably due to the surface adsorbates. Based on the Sn K-edge FT-EXAFS spectra measured in CEY mode (Fig. 2i), such oxidized Sn species might come from a small amount of SnO_2 at the surface.

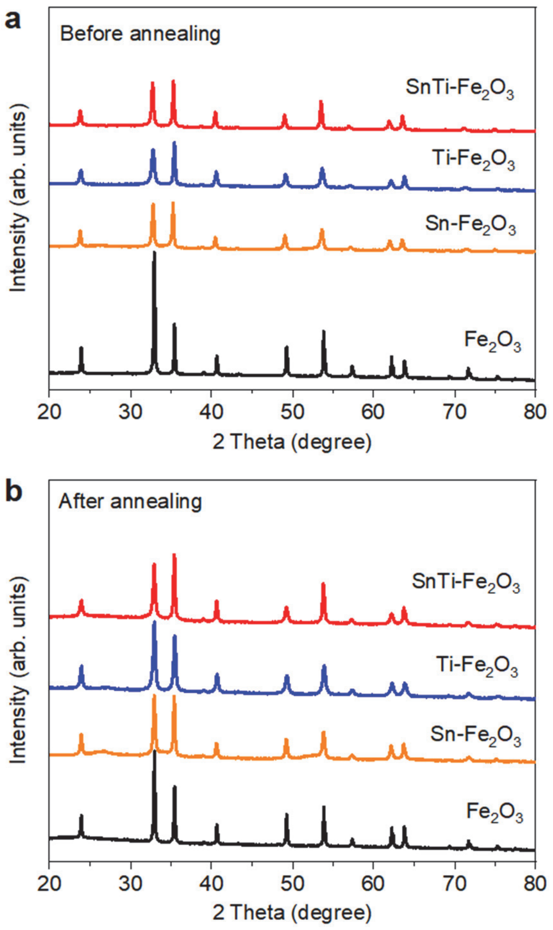

Supplementary Fig. 9. Powder XRD measurements. XRD patterns for the as-synthesized samples (before annealing) (**a**) and annealed samples (**b**) measured with a scaling rate of 10° min^{-1}.

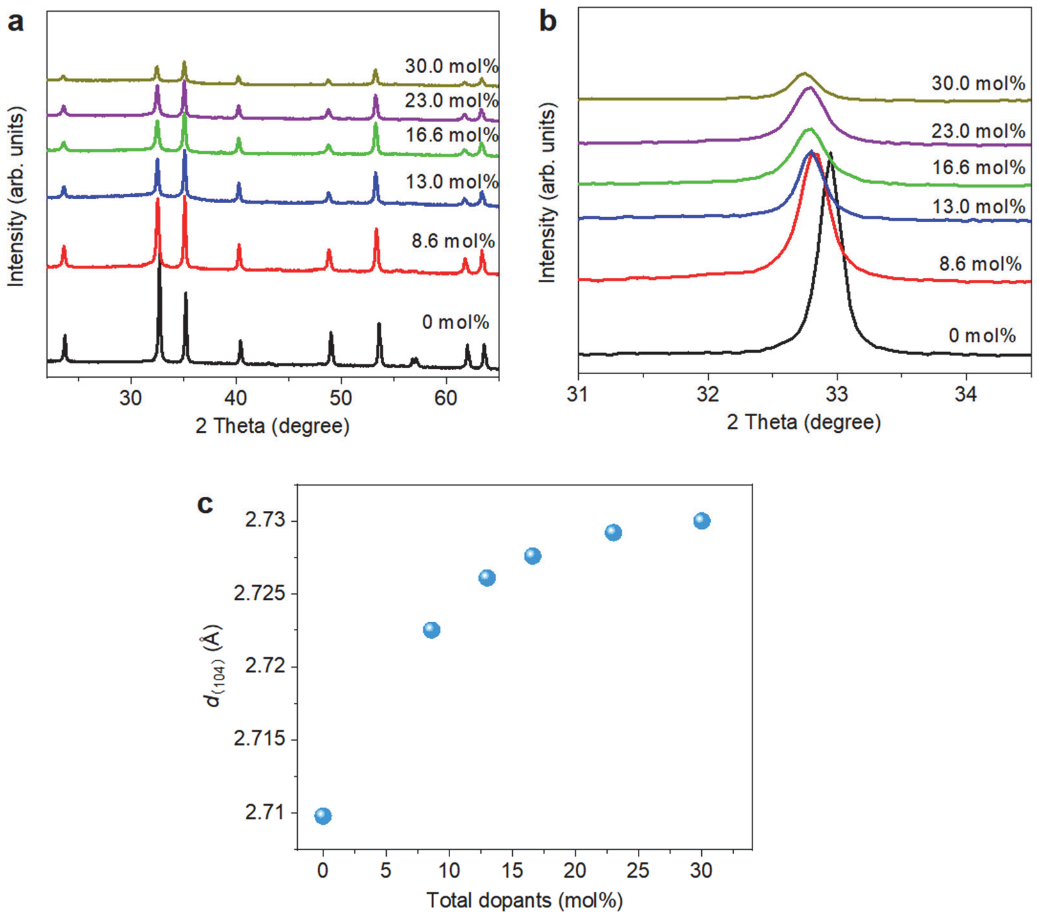

Supplementary Fig. 10. Analysis of XRD data. a, XRD patterns of the as-synthesized SnTi-Fe_2O_3 with different dopant concentrations. **b**, Enlarged view in the range of 31–34.5°. **c**, Lattice spacing d_{104} values of the samples.

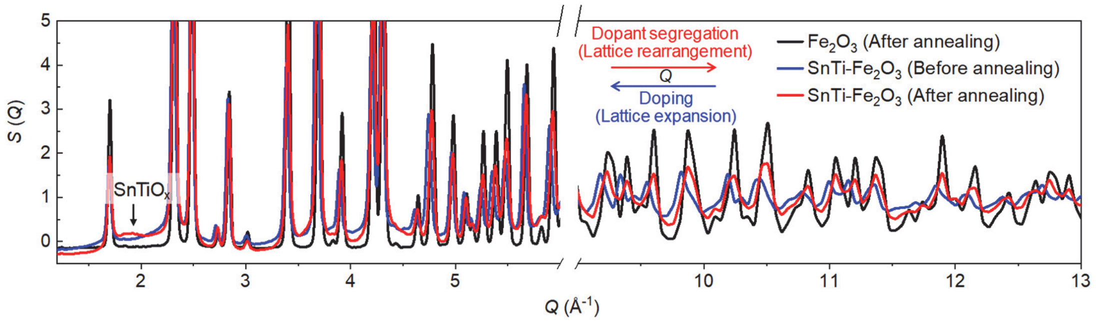

Supplementary Fig. 11. X-ray structure factor profiles of the samples.

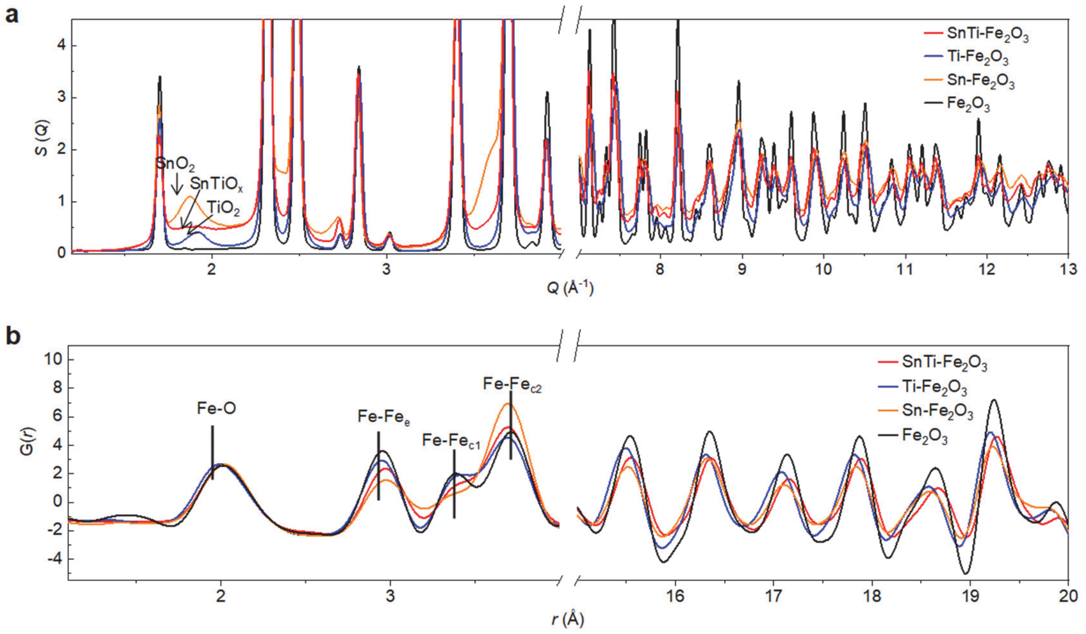

Supplementary Fig. 12. X-ray total scattering and PDF analysis. Structure factor profiles (**a**) and the corresponding pair distribution functions (PDFs) (**b**) of the samples after thermal treatment.

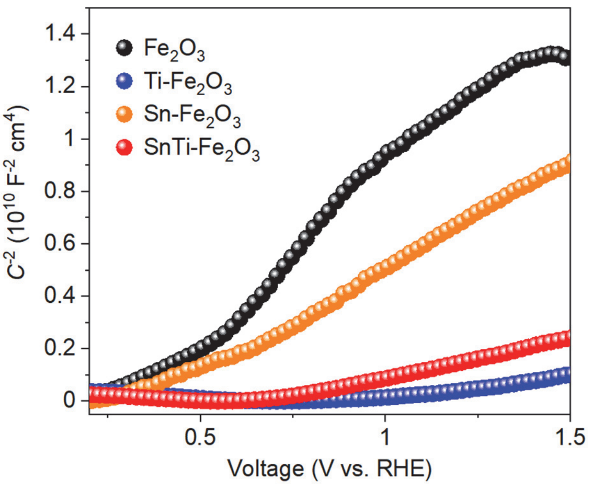

Supplementary Fig. 13. Mott-Schottky plots of the prepared photoanodes. The carrier densities were determined from the slopes of the plots (Supplementary Table 1).

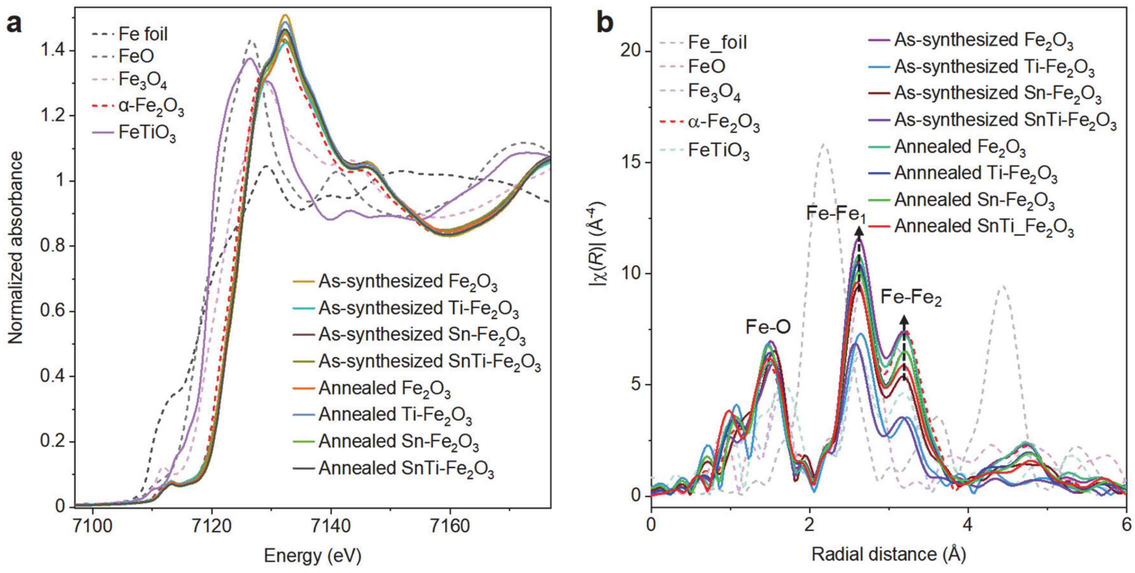

Supplementary Fig. 14. XAS measurements. Fe K-edge XANES spectra (**a**) and the corresponding FT-EXAFS spectra (**b**) of the prepared MC samples and reference samples. All the hematite-based MC samples show the same XANES spectra of α-Fe_2O_3. The FT-EXAFS spectra of the as-synthesized doped MC samples show lower intensities of Fe-Fe_1 and Fe-Fe_2 edge peaks due to the replacement with dopant ions (Sn^{2+} or Ti^{4+}), thus leading to the slight shift of these peaks. After the annealing treatment, these peaks are corresponding well to the pure hematite, indicating that the majority of the dopants diffused out from the hematite lattice.

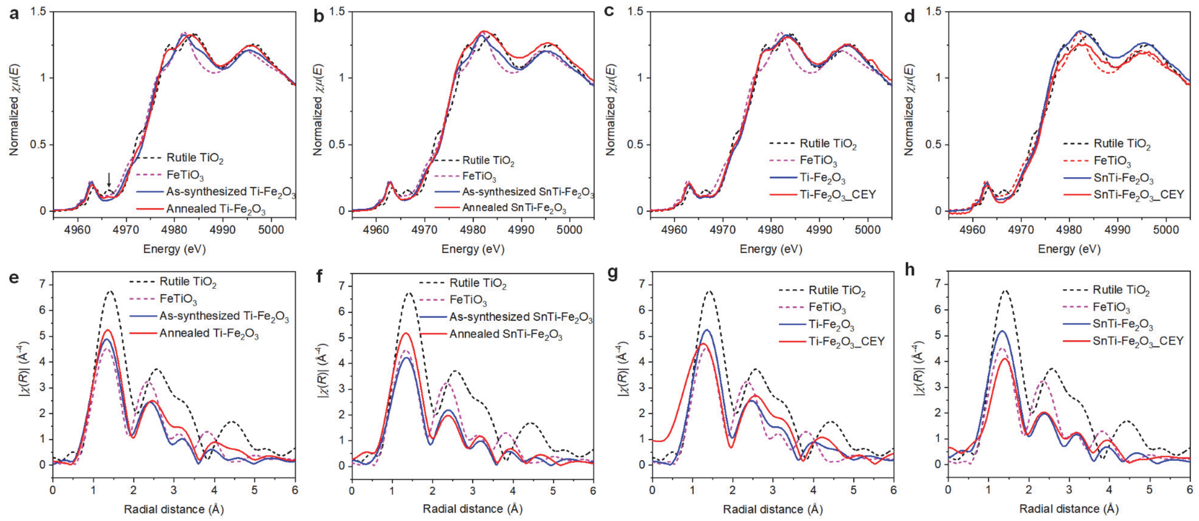

Supplementary Fig. 15. Analysis of Ti K-edge XAS data. a, Ti K-edge XANES spectra of as-synthesized Ti-Fe_2O_3 and annealed Ti-Fe_2O_3, and (**e**) their corresponding FT-EXAFS spectra. **b**, Ti K-edge XANES spectra of as-synthesized SnTi-Fe_2O_3 and annealed SnTi-Fe_2O_3, and their corresponding FT-EXAFS spectra (**f**). **c**, Ti K-edge XANES spectra of annealed Ti-Fe_2O_3 measured in transmission and CEY modes, and (**g**) their corresponding FT-EXAFS spectra. **d**, Ti K-edge XANES spectra of annealed SnTi-Fe_2O_3 measured in transmission and CEY modes, and their corresponding FT-EXAFS spectra (**h**). The Ti K-edge XANES spectrum line shape of both the as-synthesized Ti-Fe_2O_3 and SnTi-Fe_2O_3 is similar to that of the $FeTiO_3$ sample with the strongest third post-edge absorption at a similar position (Supplementary Figs. 15a and b), indicating the similar oxidation environment of Ti ions due to the doping of Ti^{4+} in hematite. For the annealed Ti-Fe_2O_3, a characteristic small pre-edge peak of rutile (as indicated by the arrow) was detected, and the line shape and absorption peaks in the post-edge region are also very close to that of rutile (Supplementary Fig. 15a and b), indicating the formation of rutile phase overlayer in this sample. This was further proven by the corresponding Ti K-edge EXAFS spectrum which shows the same Ti-coordination as that of rutile (Supplementary Figs. 15e and g). For the annealed SnTi-Fe_2O_3 sample, no characteristic small pre-edge peak of rutile was detected and the line shape and post-edge absorption peaks were also different from those of rutile (Supplementary Figs. 15b and d), suggesting the absence of rutile phase in this sample. In addition, the first post-edge absorption peak is becoming stronger and smoother compared to that of $FeTiO_3$, which is due to the formation of Sn–Ti coordination, as indicated by the corresponding Ti K-edge FT-EXAFS spectrum which show a Sn–Ti shell located between the peaks close to those reported for Fe–Ti shell of $FeTiO_3$ and Sn–Sn shell of SnO_2 (Supplementary Figs. 15f and h).

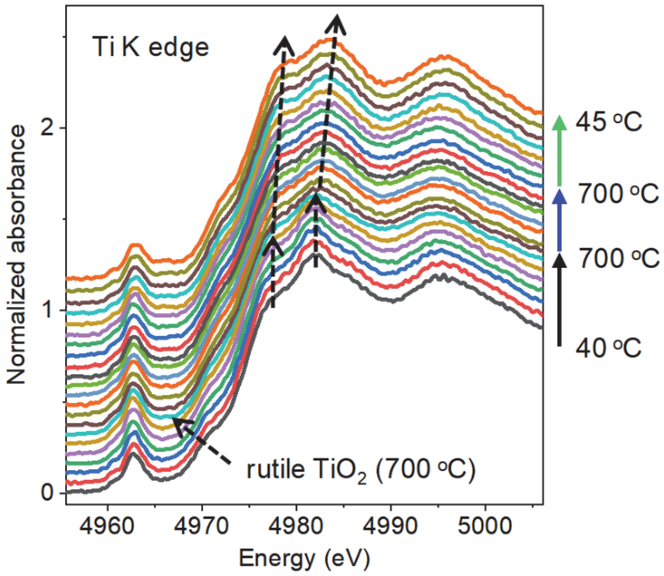

Supplementary Fig. 16. In situ Ti K-edge XANES spectra of as-synthesized Ti-Fe_2O_3. The heating of the sample was performed with the same heating procedure as the electrode preparation. The spectra are shifted along the y axis for the sake of better clarity. When the temperature was increased from 40 to 700 °C, a negligible change of peak shape and position was observed for both the samples. However, the characteristic peak of rutile phase appeared and the strongest second post-edge peak started shift to higher energies when the temperature reached 700 °C. Simultaneously, the intensity of first post-edge peak became stronger with further increasing heating time at 700 °C, and second post-edge peak significantly shift to larger energies, revealing the thermal-induced segregation of Ti ions followed by oxidation to form TiO_2 in rutile phase.

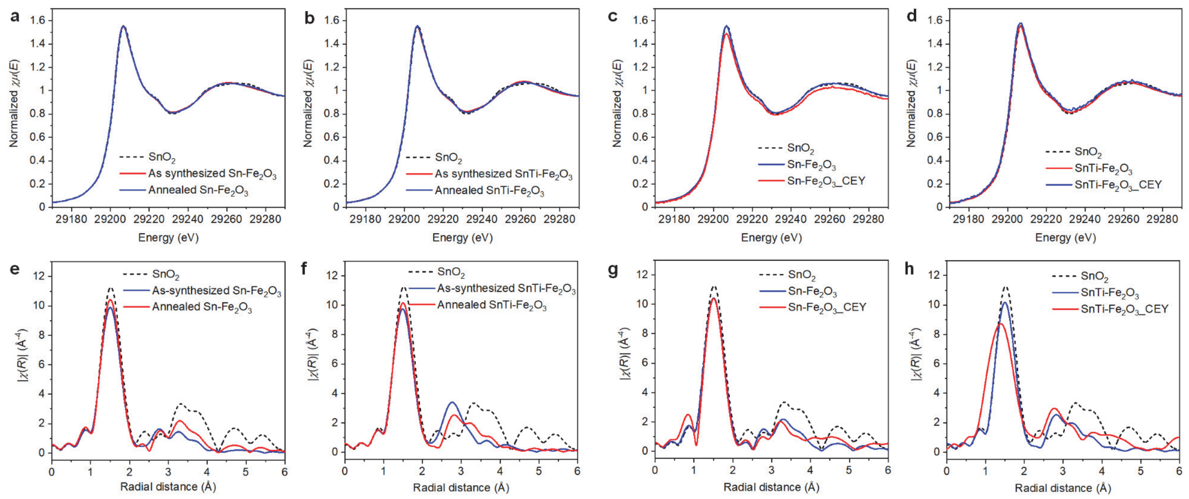

Supplementary Fig. 17. Analysis of Sn K-edge XAS data. a, Sn K-edge XANES spectra of as-synthesized $Sn-Fe_2O_3$ and annealed $Sn-Fe_2O_3$, and their corresponding FT-EXAFS spectra (**e**). **b**, Sn K-edge XANES spectra of as-synthesized $SnTi-Fe_2O_3$ and annealed $SnTi-Fe_2O_3$, and their corresponding FT-EXAFS spectra (**f**). **c**, Sn K-edge XANES spectra of annealed $Sn-Fe_2O_3$ measured in transmission mode and CEY mode, and their corresponding FT-EXAFS spectra (**g**). **d**, Sn K-edge XANES spectra of annealed $SnTi-Fe_2O_3$ measured in transmission and CEY modes, and their corresponding FT-EXAFS spectra (**h**). The Sn K-edge EXAFS spectra of both the as-synthesized $Sn-Fe_2O_3$ and $SnTi-Fe_2O_3$ show the main shell at 2.76 Å (Supplementary Figs. 17e and f), which can be assigned to the Sn–Fe coordination due to the replacement of Fe^{3+} ions with Sn^{2+} ions in hematite (i.e. doping). The peak position of Sn–Sn for the annealed $Sn-Fe_2O_3$ is the same as that of the reference SnO_2 sample (Fig. 4d), proving the formation of a SnO_2 overlayer. For the annealed $SnTi-Fe_2O_3$, the Sn–Sn bonding has a shorter radial distance than that of SnO_2, which is due to the formation of Sn–Ti coordination (i.e., $SnTiO_{3-x}$).[23]

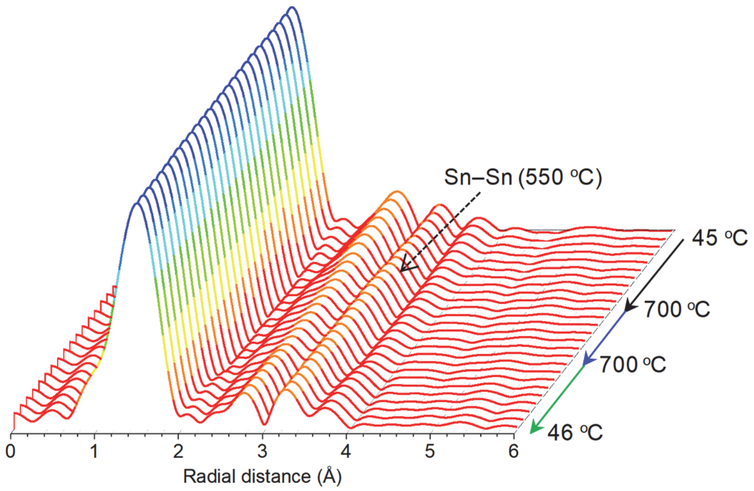

Supplementary Fig. 18. In situ Sn K-edge FT-EXAFS spectra of as-synthesized Sn-Fe_2O_3. The heating of the sample was performed with the same heating procedure as the electrode preparation.

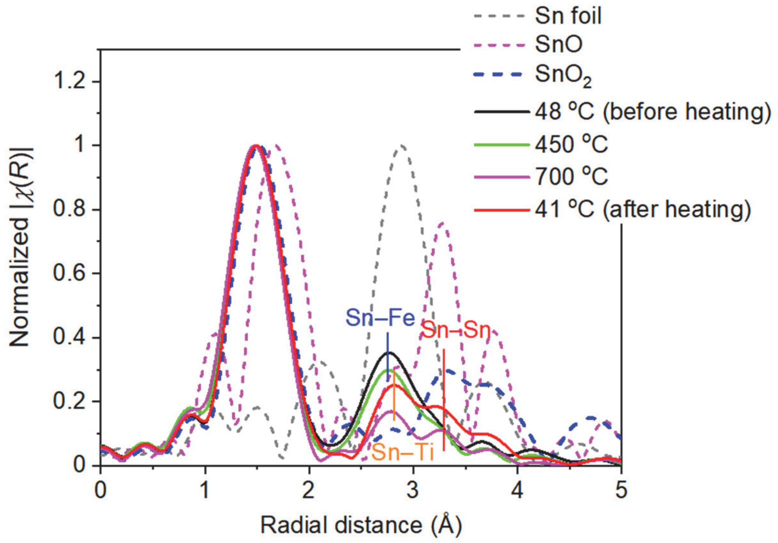

Supplementary Fig. 19. Sn K-edge FT-EXAFS spectra. The data were obtained from in situ measurements of SnTi-Fe_2O_3 (see Fig. 4c). The spectra of the reference samples are also shown. Because the phase transformation of SnO into SnO_2 occurs above 420 °C in air,[24] the spectrum at 450 °C would be a superposition of the spectra of SnO and SnO_2. However, a complete structural assignment of intermediates (including Sn–Fe oxides) is difficult due to weak signal intensities and lack of appropriate reference compounds.

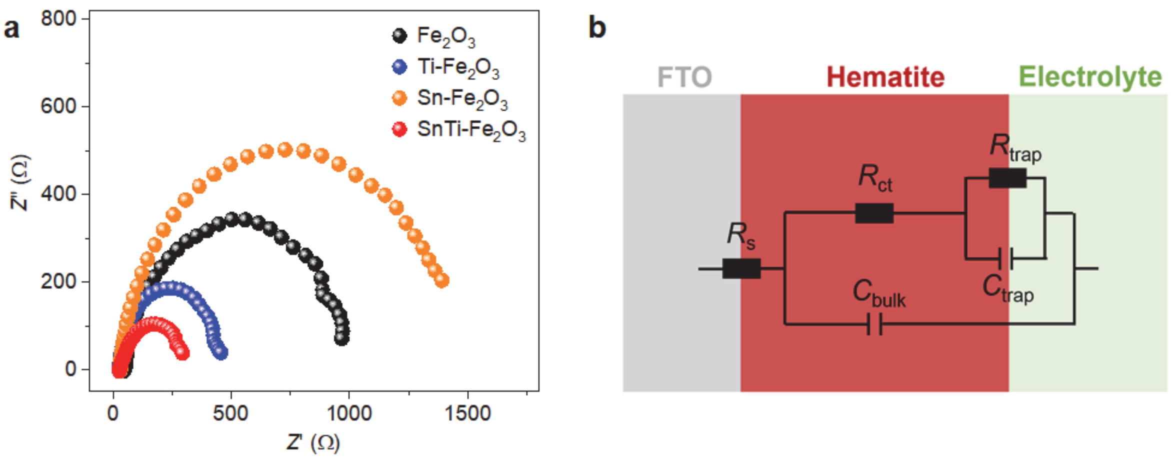

Supplementary Fig. 20. EIS analysis. a, EIS plots of the samples measured at 1.23 V vs. RHE under back illumination. **b**, The equivalent circuit model used to simulate the EIS data.

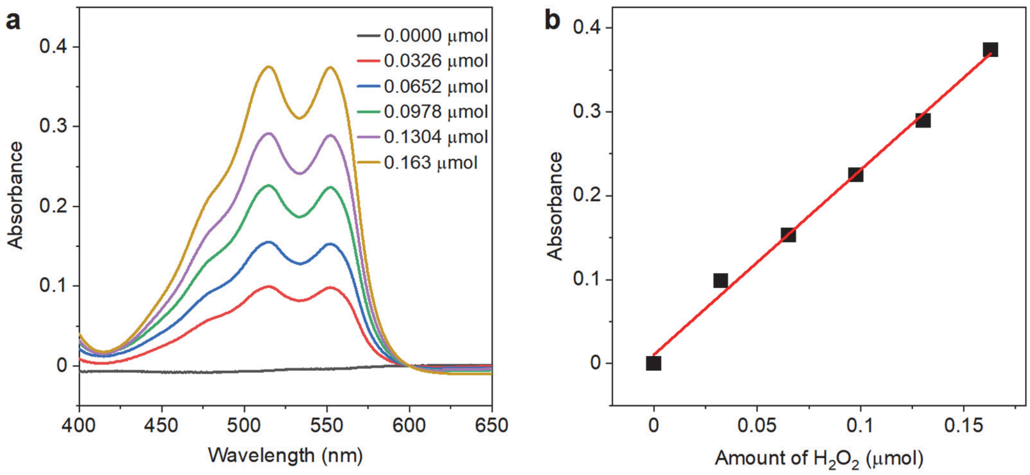

Supplementary Fig. 21. Detection of H_2O_2. a, Optical absorption spectra obtained using standard H_2O_2. **b**, The calibration curve between the absorbance and the amount of H_2O_2.

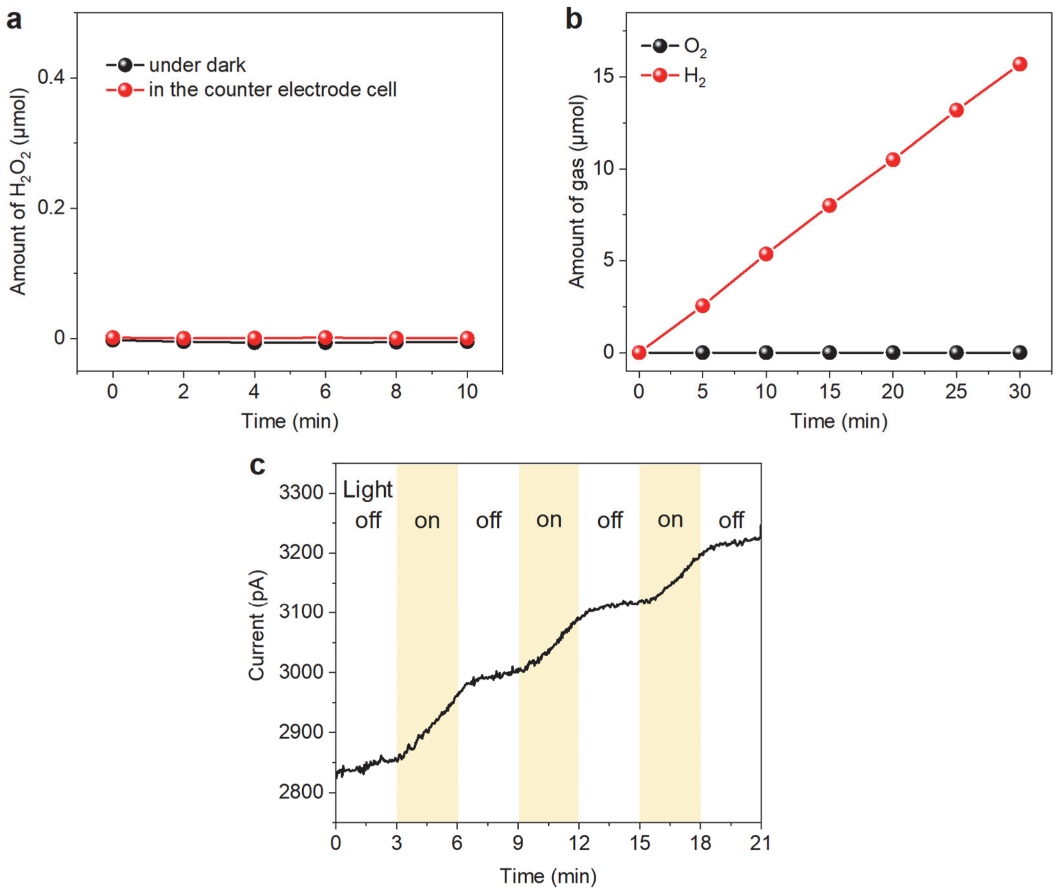

Supplementary Fig. 22. Product analysis. a, H_2O_2 generation from a SnTi-Fe_2O_3 photoanode under dark (black) and in the Pt counter electrode cell with back illumination of a SnTi-Fe_2O_3 photoanode (red) at 1.5 V vs. RHE. **b**, Gas evolved from the SnTi-Fe_2O_3 photoanode and Pt counter electrode cells under back illumination at 1.6 V vs. RHE. **c**, Electrochemical detection of dissolved O_2 in 1.0 M $NaHCO_3$ in the SnTi-Fe_2O_3 photoanode cell under dark and back illumination with AM 1.5 G simulated sunlight at 1.6 V vs. RHE. Before the measurements, the electrolyte was bubbled with Ar gas until the current value becomes constant. The FE of O_2 evolution was estimated to be approximately 20%. This value would be reasonable when considering the experimental error in the H_2O_2 analysis.

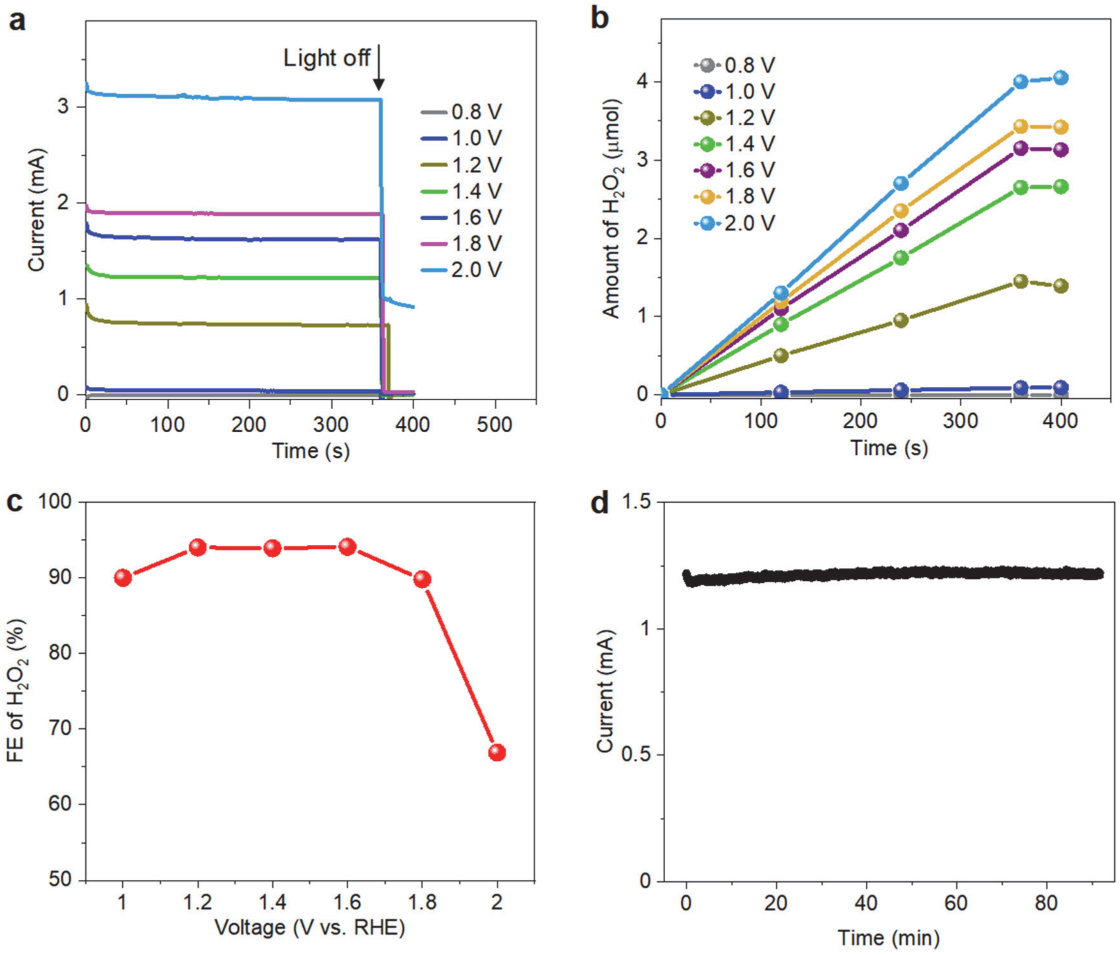

Supplementary Fig. 23. Generation of H_2O_2 under different conditions. Current–time curves (**a**) and corresponding amounts of H_2O_2 (**b**) generated at different applied voltages. **c**, The voltage-depended FEs of H_2O_2 by a SnTi-Fe_2O_3 photoanode. **d**, Current–time curve of a SnTi-Fe_2O_3 photoanode measured at 1.5 V vs. RHE.

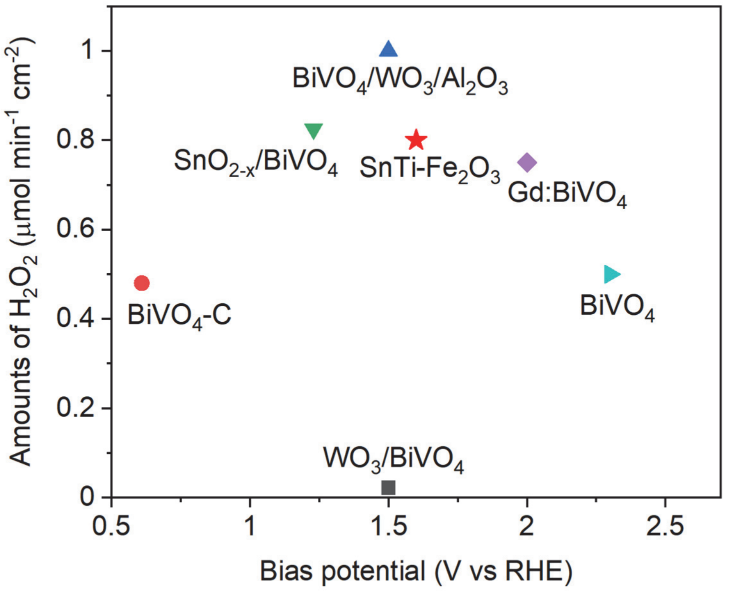

Supplementary Fig. 24. PEC H_2O_2 production activity. The performance of the optimized SnTi-Fe_2O_3 photoanode is compared with the reported photoanodes.[12,15,17,25–27]

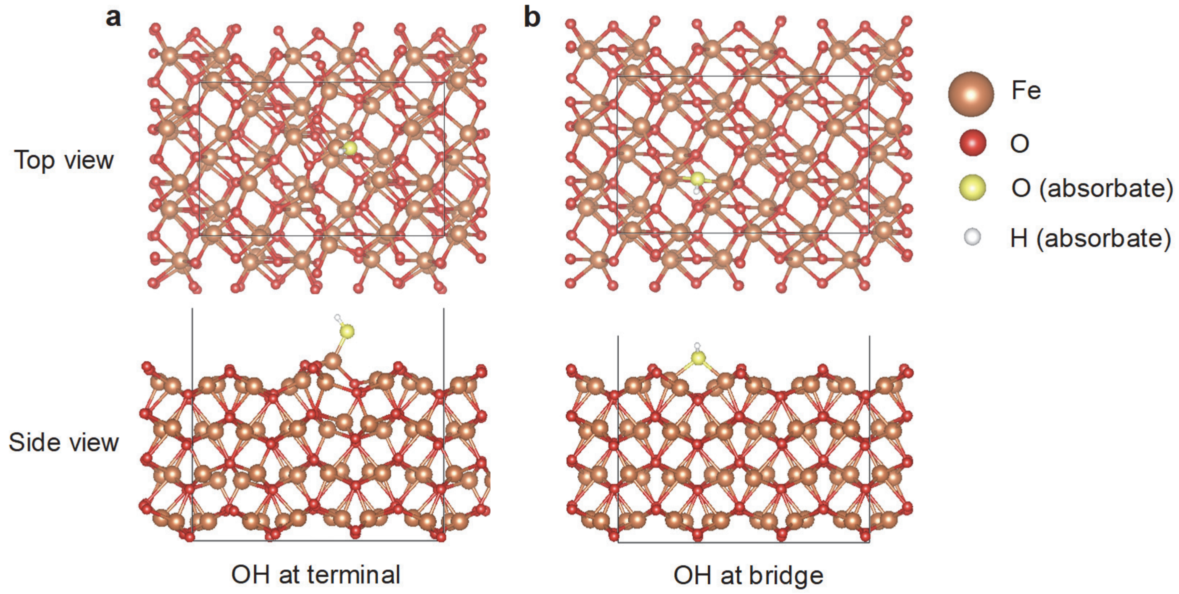

Supplementary Fig. 25. Structures modelled for hematite. Hematite (110) surfaces with OH at the terminal site (**a**) and at the bridge site (**b**).

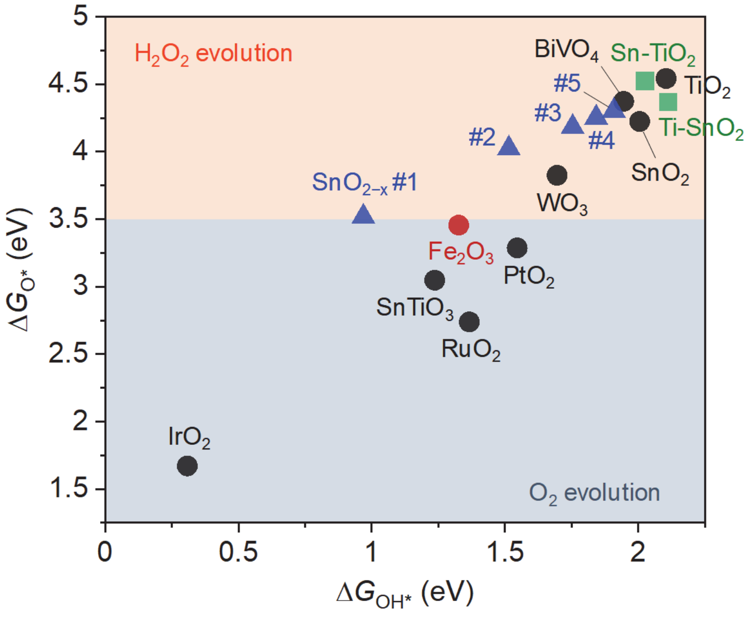

Supplementary Fig. 26. Product selectivity diagram in terms of ΔG_{OH*} and ΔG_{O*}. Blue and red highlighted colours indicate the regions in which O_2 and H_2O_2 are expected to be the major product, respectively, on the basis of purely thermodynamic considerations. H_2O_2 synthesis is favoured when ΔG_{OH*} is between 1.6 and 2.4 eV and ΔG_{O*} is larger than 3.5 eV.[15,28]

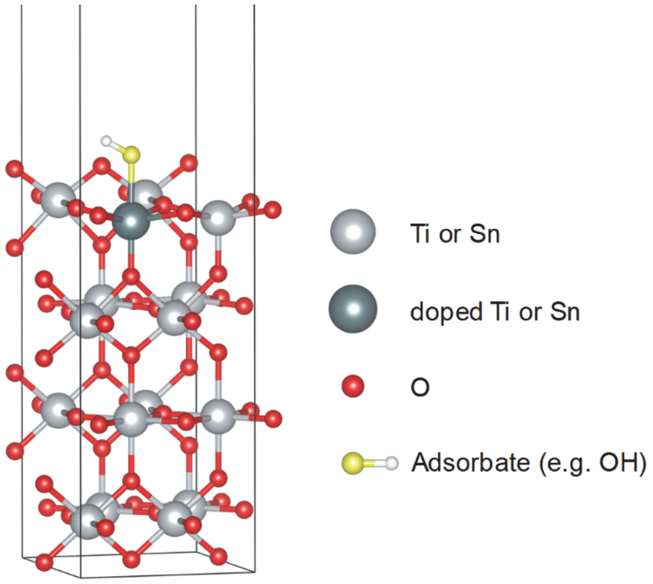

Supplementary Fig. 27. Structure modelled for Sn^{4+}-doped TiO_2 and Ti^{4+}-doped SnO_2. The dopants are considered as the surface active sites.

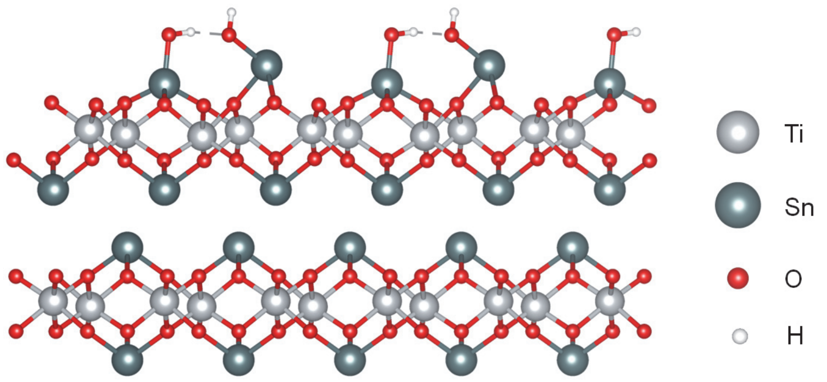

Supplementary Fig. 28. Structure modelled for ilmenite-type $SnTiO_3$ (1/3 ML). Each Sn^{2+} possesses a lone pair, forming layers separated by a van der Waals gap.[19]

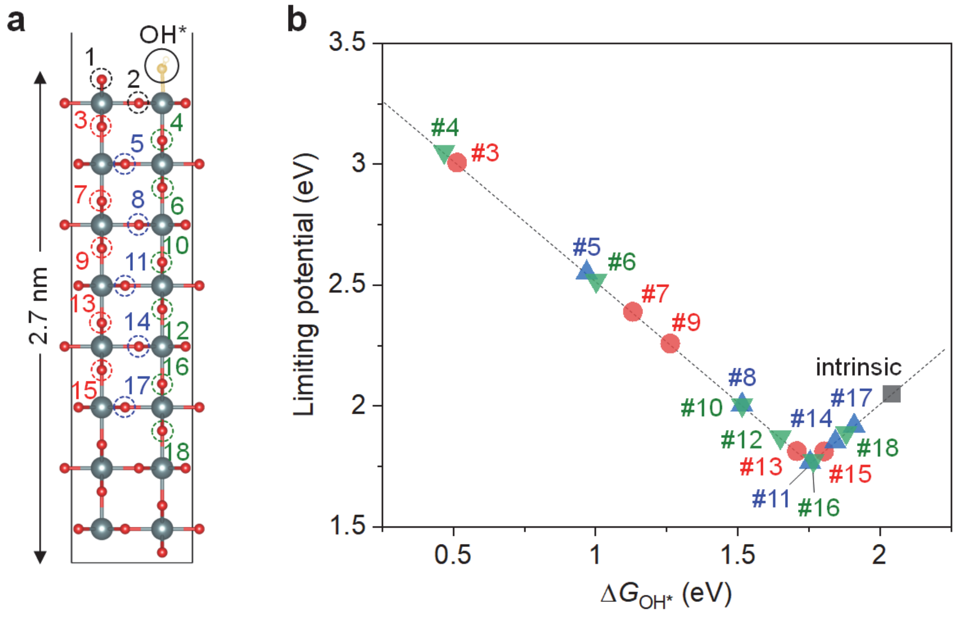

Supplementary Fig. 29. DFT calculations of SnO_{2-x}. a, Structure model of SnO_{2-x} with V_O. **b**, Activity volcano plots based on calculated limiting potentials as a function of ΔG_{OH^*}. ΔG values were not determined for SnO_{2-x} with V_O at the positions 1 and 2 because of the oxidation of V_O sites by OH*.

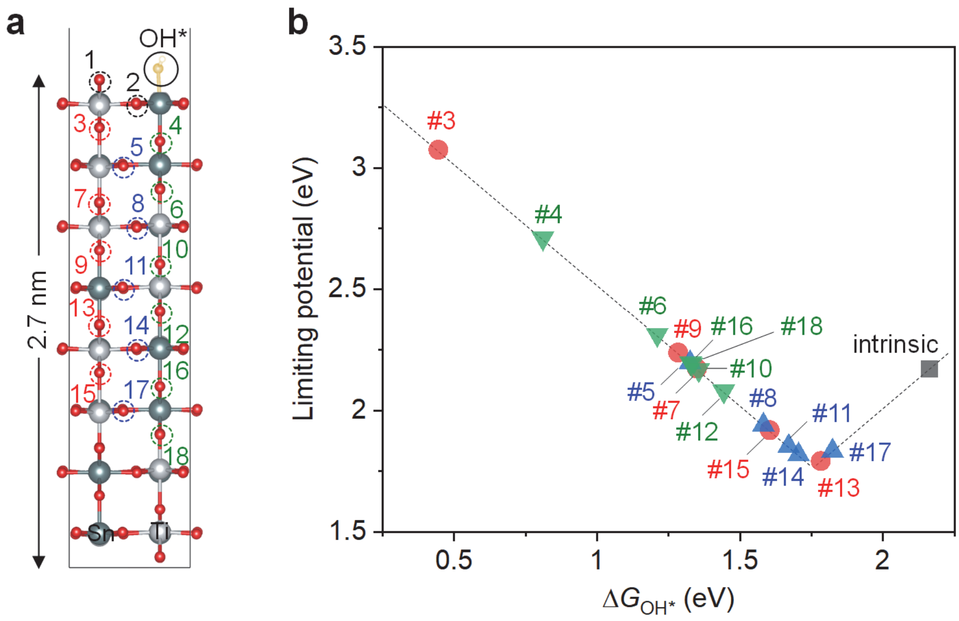

Supplementary Fig. 30. DFT calculations of $Sn_{0.5}Ti_{0.5}O_{2-x}$. a, Structure model of $Sn_{0.5}Ti_{0.5}O_{2-x}$ with V_O.[23] **b**, Activity volcano plots based on calculated limiting potentials as a function of ΔG_{OH*}. ΔG values were not determined for $Sn_{0.5}Ti_{0.5}O_{2-x}$ with V_O at the positions 1 and 2 because of the oxidation of V_O sites by OH*.

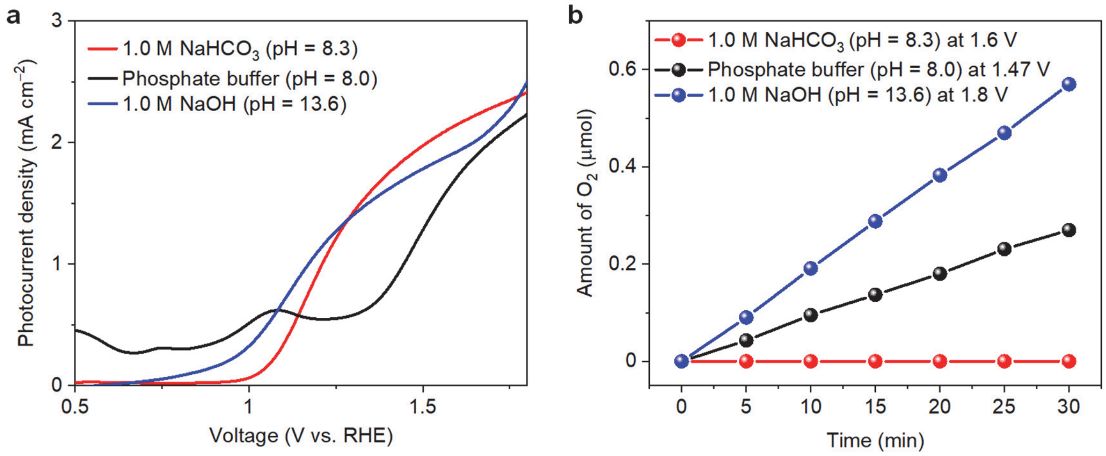

Supplementary Fig. 31. Effects of electrolytes. a, Current density–voltage curves of SnTi-Fe_2O_3 photoanodes measured in different electrolytes. **b**, Gas evolved from SnTi-Fe_2O_3 photoanodes measured in different electrolytes.

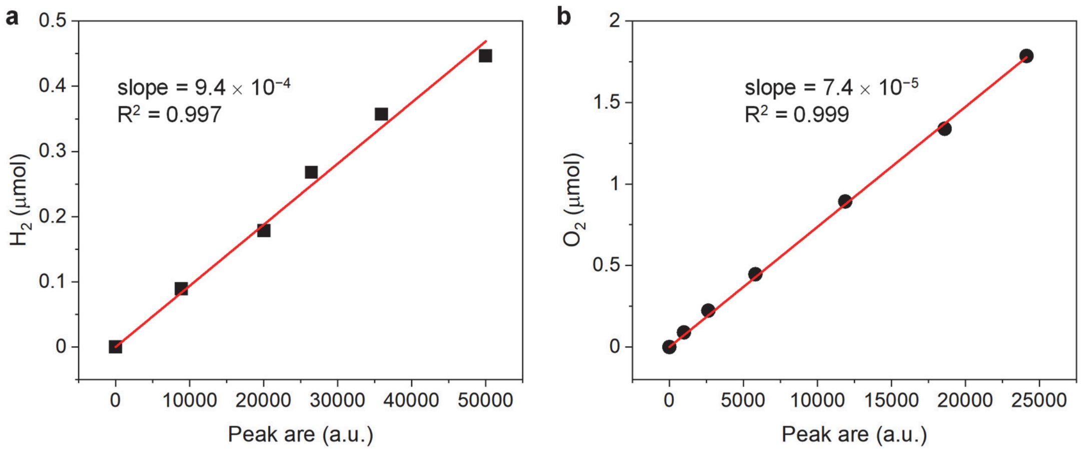

Supplementary Fig. 32. Calibration curves. a, The calibration curve between the peak area and amount of H_2. **b**, The calibration curve between the peak area and amount of O_2.

Supplementary Tables

Supplementary Table 1. Parameters Determined from Electrochemical Measurements and Carrier Densities Calculated According to the Slopes of the Mott-Schottky Curves

Electrodes	R_s (Ω cm^2)	R_{ct} (Ω cm^2)	R_{trap} (Ω cm^2)	Carrier density [a] (cm^{-3})	W [a,b] (nm)
Fe_2O_3	100	1324	2360	(1.1 ± 0.1) × 10^{20}	8.7
Ti-Fe_2O_3	62	346	454	(4.6 ± 0.1) × 10^{20}	4.3
Sn-Fe_2O_3	71	829	2787	(1.2 ± 0.1) × 10^{20}	8.5
SnTi-Fe_2O_3	61	361	328	(3.7 ± 0.1) ×10^{20}	4.8

[a] See Supplementary Note 2 for details.

[b] Calculated using the bias potential of 1.23 V vs. RHE.

Supplementary Table 2. ΔG_{OH*} and ΔG_{O*} Calculated for Fe_2O_3

Location and number of adsorbate	x/12 ML	ΔG_{OH*} (eV)	ΔG_{O*} (eV)	$\Delta\Delta G_{OH*}$ (eV)
Terminal 1	1	1.33	3.45	–
Terminal 2	2	1.74	3.66	2.15
Terminal 3	3	1.88	3.78	2.16
Terminal 3 + Bridge 1	4	1.78	3.96	1.48
Terminal 3 + Bridge 2	5	1.80	4.15	1.88
Terminal 3 + Bridge 3	6	1.78	3.98	1.68

Supplementary Table 3. Computed ΔG_{OH*} and ΔG_{O*} for Representative Oxides

Oxide	ΔG_{OH*} (eV) [a]	ΔG_{O*} (eV) [a]
TiO_2	2.11	4.54
Sn^{4+}-doped TiO_2	2.03	4.52
SnO_2	2.01	4.22
Ti^{4+}-doped SnO_2	2.12	4.36
$SnTiO_3$ (1/3 ML)	1.24	3.04
Fe_2O_3 (1/12 ML, Terminal)	1.33	3.45
IrO_2	0.31	1.66
RuO_2	1.37	2.73
PtO_2	1.55	3.28
WO_3	1.70	3.82
$BiVO_4$	1.95	4.37

[a] For IrO_2, RhO_2, and PtO_2, the values were taken from Ref. [29]. The values for WO_3 and $BiVO_4$ were adapted from Refs. [30] and [28].

Supplementary Table 4. Computed ΔG_{OH^*} and ΔG_{O^*} for SnO_{2-x}

Vo site index [a]	ΔG_{OH^*} (eV)	ΔG_{O^*} (eV)
(Intrinsic)	2.05	4.40
3	0.52	2.91
4	0.47	3.01
5	0.97	3.51
6	1.00	3.56
7	1.13	3.65
8	1.52	4.02
9	1.27	3.93
10	1.52	3.98
11	1.76	4.18
12	1.65	4.14
13	1.71	4.12
14	1.85	4.25
15	1.81	4.21
16	1.77	4.18
17	1.91	4.30
18	1.89	4.27

[a] The index corresponds to that of Supplementary Fig. 29.

Supplementary Table 5. Computed ΔG_{OH*} and ΔG_{O*} for $Sn_{0.5}Ti_{0.5}O_{2-x}$

Vo site index [a]	ΔG_{OH*} (eV)	ΔG_{O*} (eV)
(Intrinsic)	2.17	4.40
3	0.45	2.64
4	0.81	3.24
5	1.33	3.87
6	1.21	3.78
7	1.35	3.88
8	1.59	4.09
9	1.29	3.80
10	1.36	3.91
11	1.67	4.17
12	1.45	3.94
13	1.79	4.32
14	1.71	4.23
15	1.61	4.18
16	1.33	3.90
17	1.83	4.29
18	1.34	3.90

[a] The index corresponds to that of Supplementary Fig. 30.

Supplementary References

1. Kliewer, K. L. & Koehler, J. S. Space Charge in Ionic Crystals. I. General Approach with Application to NaCl. *Phys. Rev.* **140**, A1226–A1240 (1965).
2. Yoon, H. I. *et al.* Probing dopant segregation in distinct cation sites at perovskite oxide polycrystal interfaces. *Nat. Commun.* **8**, 1417 (2017).
3. Chung, S.-Y., Choi, S.-Y., Yoon, H.-I., Kim, H.-S. & Bae, H. Bin. Subsurface Space-Charge Dopant Segregation to Compensate Surface Excess Charge in a Perovskite Oxide. *Angew. Chem. Int. Ed.* **55**, 9680–9684 (2016).
4. De Souza, R. A. The formation of equilibrium space-charge zones at grain boundaries in the perovskite oxide $SrTiO_3$. *Phys. Chem. Chem. Phys.* **11**, 9939 (2009).
5. Scherrer, B. *et al.* Defect Segregation and Its Effect on the Photoelectrochemical Properties of Ti-Doped Hematite Photoanodes for Solar Water Splitting. *Chem. Mater.* **32**, 1031–1040 (2020).
6. Bosman, A. J. & van Daal, H. J. Small-polaron versus band conduction in some transition-metal oxides. *Adv. Phys.* **19**, 1–117 (1970).
7. Liao, P., Toroker, M. C. & Carter, E. A. Electron Transport in Pure and Doped Hematite. *Nano Lett.* **11**, 1775–1781 (2011).
8. Carneiro, L. M. *et al.* Excitation-wavelength-dependent small polaron trapping of photoexcited carriers in α-Fe_2O_3. *Nat. Mater.* **16**, 819–825 (2017).
9. Zhang, Z., Nagashima, H. & Tachikawa, T. Ultra-Narrow Depletion Layers in a Hematite Mesocrystal-Based Photoanode for Boosting Multihole Water Oxidation. *Angew. Chem. Int. Ed.* **59**, 9047–9054 (2020).
10. Xu, Z., Rossmeisl, J. & Kitchin, J. R. A Linear Response DFT+*U* Study of Trends in the Oxygen Evolution Activity of Transition Metal Rutile Dioxides. *J. Phys. Chem. C* **119**, 4827–4833 (2015).
11. Zhang, X., Klaver, P., van Santen, R., van de Sanden, M. C. M. & Bieberle-Hütter, A. Oxygen Evolution at Hematite Surfaces: The Impact of Structure and Oxygen Vacancies on Lowering the Overpotential. *J. Phys. Chem. C* **120**, 18201–18208 (2016).
12. Fuku, K. & Sayama, K. Efficient oxidative hydrogen peroxide production and accumulation in photoelectrochemical water splitting using a tungsten trioxide/bismuth vanadate photoanode. *Chem. Commun.* **52**, 5406–5409 (2016).
13. Fuku, K., Miyase, Y., Miseki, Y., Gunji, T. & Sayama, K. WO_3/$BiVO_4$ photoanode coated with mesoporous Al_2O_3 layer for oxidative production of hydrogen peroxide from water with high selectivity. *RSC Adv.* **7**, 47619–47623 (2017).
14. Patra, S. G., Mizrahi, A. & Meyerstein, D. The Role of Carbonate in Catalytic Oxidations. *Acc. Chem. Res.* **53**, 2189–2200 (2020).
15. Shi, X. *et al.* Understanding activity trends in electrochemical water oxidation to form hydrogen peroxide. *Nat. Commun.* **8**, 701 (2017).
16. Liu, J., Zou, Y., Jin, B., Zhang, K. & Park, J. H. Hydrogen Peroxide Production from Solar Water Oxidation. *ACS Energy Lett.* **4**, 3018–3027 (2019).
17. Baek, J. H. *et al.* Selective and Efficient Gd-Doped $BiVO_4$ Photoanode for Two-Electron Water Oxidation to H_2O_2. *ACS Energy Lett.* **4**, 720–728 (2019).
18. Gloter, A., Ewels, C., Umek, P., Arcon, D. & Colliex, C. Electronic structure of titania-based nanotubes

investigated by EELS spectroscopy. *Phys. Rev. B - Condens. Matter Mater. Phys.* **80**, 035413 (2009).
19. Diehl, L. *et al.* Structure-Directing Lone Pairs: Synthesis and Structural Characterization of $SnTiO_3$. *Chem. Mater.* **30**, 8932–8938 (2018).
20. Lorenz, H. *et al.* Preparation and structural characterization of SnO_2 and GeO_2 methanol steam reforming thin film model catalysts by (HR)TEM. *Mater. Chem. Phys.* **122**, 623–629 (2010).
21. Moreno, M. S., Egerton, R. F. & Midgley, P. A. Differentiation of tin oxides using electron energy-loss spectroscopy. *Phys. Rev. B - Condens. Matter Mater. Phys.* **69**, 2–5 (2004).
22. Dennenwaldt, T. *et al.* Insights into the structural, electronic, and magnetic properties of $Fe_{2-x}Ti_xO_3/Fe_2O_3$ thin films with x = 0.44 grown on Al_2O_3 (0001). *J. Mater. Sci.* **50**, 122–137 (2015).
23. Chen, Y. C. *et al.* Rutile-type $(Ti,Sn)O_2$ nanorods as efficient anode materials toward its lithium storage capabilities. *Nanoscale* **5**, 2254–2258 (2013).
24. Xia, W. *et al.* High-efficiency photocatalytic activity of type II SnO/Sn_3O_4 heterostructures via interfacial charge transfer. *CrystEngComm* **16**, 6841–6847 (2014).
25. Fuku, K., Miyase, Y., Miseki, Y., Gunji, T. & Sayama, K. $WO_3/BiVO_4$ photoanode coated with mesoporous Al_2O_3 layer for oxidative production of hydrogen peroxide from water with high selectivity. *RSC Adv.* **7**, 47619–47623 (2017).
26. Zhang, K. *et al.* Near-complete suppression of oxygen evolution for photoelectrochemical H_2O oxidative H_2O_2 synthesis. *J. Am. Chem. Soc.* **142**, 8641–8648 (2020).
27. Shi, X., Zhang, Y., Siahrostami, S. & Zheng, X. Light-Driven $BiVO_4$–C Fuel Cell with Simultaneous Production of H_2O_2. *Adv. Energy Mater.* **8**, 1801158 (2018).
28. Siahrostami, S., Li, G.-L., Viswanathan, V. & Nørskov, J. K. One- or Two-Electron Water Oxidation, Hydroxyl Radical, or H_2O_2 Evolution. *J. Phys. Chem. Lett.* **8**, 1157–1160 (2017).
29. Man, I. C. *et al.* Universality in Oxygen Evolution Electrocatalysis on Oxide Surfaces. *ChemCatChem* **3**, 1159–1165 (2011).
30. Siahrostami, S., Björketun, M. E., Strasser, P., Greeley, J. & Rossmeisl, J. Tandem cathode for proton exchange membrane fuel cells. *Phys. Chem. Chem. Phys.* **15**, 9326 (2013).
